# Progress towards Eradication of Peste des Petits Ruminants through Vaccination

**DOI:** 10.3390/v13010059

**Published:** 2021-01-05

**Authors:** Hang Zhao, Felix Njeumi, Satya Parida, Camilla T. O. Benfield

**Affiliations:** 1 Jiangsu Key Laboratory for Food Quality and Safety–State Key Laboratory Cultivation Base of Ministry of Science and Technology, Jiangsu Academy of Agricultural Sciences, Nanjing 210014, China; dongyizhaohang@163.com; 2 Food and Agriculture Organization of the United Nations (FAO), Viale delle Terme di Caracalla, 00153 Rome, Italy; felix.njeumi@fao.org; 3 The Pirbright Institute, Woking GU24 0NF, UK; 4 Royal Veterinary College, University of London, London NW1 0TU, UK

**Keywords:** peste des petits ruminants, eradication, vaccination campaign, vaccine, surveillance, global eradication program (GEP)

## Abstract

Peste des petits ruminants (PPR) is a transboundary viral disease that threatens more than 1.74 billion goats and sheep in approximately 70 countries globally. In 2015, the international community set the goal of eradicating PPR by 2030, and, since then, Food and Agriculture Organization of the United Nations (FAO) and World Organization for Animal Health (OIE) have jointly developed and implemented the Global Control and Eradication Strategy for PPR. Here, data from the United Nations Food and Agriculture Organization Statistical Database (FAOSTAT), the OIE World Animal Health Information System (WAHIS), Regional Roadmap Meetings, and countries’ responses to PPR Monitoring and Assessment Tool (PMAT) questionnaires were analyzed to inform on current progress towards PPR eradication. OIE recorded the use of over 333 million doses of vaccine in 12 countries from 2015 to 2018, 41.8% of which were used in Asia and 58.2% in Africa. Between 2015 and 2019, a total of 12,757 PPR outbreaks were reported to OIE: 75.1% in Asia, 24.8% in Africa, and 0.1% in Europe. The number of global outbreaks in 2019 fell to 1218, compared with 3688 in 2015. Analysis of vaccine use and PPR outbreaks in countries indicates that disease control strategies, particularly vaccination campaigns and vaccine distribution strategies, still require scientific evaluation. It is imperative that vaccination is undertaken based on the epidemiology of the disease in a region and is coordinated between neighboring countries to restrict transboundary movements. Strengthening surveillance and post-vaccination sero-monitoring at the national level is also essential. The PPR vaccine stock/bank established by FAO, OIE, and other partners have improved the quality assurance and supply of vaccines. However, to achieve PPR eradication, filling the funding gap for vaccination campaigns and other program activities will be critical.

## 1. Introduction

Peste des petits ruminants (PPR) is a highly contagious transboundary disease caused by the Peste des petits ruminants virus (PPRV) [1,2,3]. PPRV belongs to the *morbillivirus* genus, *Paramyxoviridae* family [4] and has been renamed as small ruminant morbillivirus (SRMV) by the International Committee on Taxonomy of Viruses in 2016 [5].

There is only one serotype of PPRV, but phylogenetic analysis based on partial N or F gene sequences groups PPRV strains into lineages I, II, III, and IV [6]. Historically, PPRV strains found in Africa belonged to lineage I and lineage II and were mainly prevalent in West Africa. Lineage III is mostly found in Arabia and recently circulating in East Africa [7,8], but has also been isolated in southern India on one occasion. Lineage IV is usually found in Asia, and hence, has been termed the Asian lineage [9]. A recent review of currently available molecular epidemiological data was carried out in Africa [10] and showed that since 2008, lineage IV has also been continually identified in Africa [9,11,12].

PPR was first described as a rinderpest (RP)-like disease of small domestic ruminants. However, in the recent-past PPRV has been reported to infect not only goats and sheep, but also camels [13], cattle [14], water buffalo [15,16], and wildlife species, including African buffalo [17,18], saiga antelope [19,20], dorcas gazelles [19,21], gemsbok [22], Nubian ibex [23], and some other wild ungulate species [19,20]. Reports of PPRV detection in Asiatic lions [24] and dogs [3,25] may reflect contamination of their food with infected ruminants, but their competence as hosts has not been established [13,26,27,28,29]. Goats and sheep are the main hosts for PPRV and play a major role in its transmission and global epidemiology. The current evidence is not sufficient to clarify whether species other than goats and sheep play a role in PPRV epidemiology, although recent experimental studies have reported that suids were able to transmit PPRV [30] while camelids and cattle may be dead-end hosts [31].

PPR is classified as a notifiable terrestrial animal disease by the World Organization for Animal Health (OIE). In 1942, PPR was first reported in the Côte d’Ivoire in West Africa. Hitherto, globally, PPR has infected 66 countries, including 38 countries in Africa, 27 countries in Asia, and one country in Europe. According to 2018 data from the United Nations Food and Agriculture Organization Statistical Database (FAOSTAT), there are a total of 2.5 billion heads of small ruminants worldwide. Of those, over 1.74 billion sheep and goats are in PPR infected countries, 56.4% (982.3 million) in Asia, 43.6% (759.1 million) in Africa, 0.1% in Europe (1.57 million). Small ruminants are the main livestock resource of more than 300 million rural households worldwide, where women play an important role in sheep and goat farming. The wide distribution of PPR across Africa, Asia, and Europe continues to damage the food security, livelihoods, and trade of herders as well as pose threats to biodiversity and ecosystem health.

In 2015, in Abidjan, Côte d’Ivoire, the Food and Agriculture Organization of the United Nations (FAO), OIE, and partners endorsed the PPR Global Control and Eradication Strategy (GCES) with the goal of global eradication of PPR by 2030. The joint FAO/OIE PPR Secretariat to support the implementation of the GCES was established in 2016. Since the autumn of 2013, OIE member countries have been able to apply to OIE for PPR free status, and by May 2014, 48 countries were officially confirmed to be free from PPR. By July 2018, a total of 56 countries and one region within Namibia were recognized as PPR free. By May 2020, Russia and Lesotho were added to the above as PPR free. To proceed from control to eradication of PPR, the GCES follows a technical sequential step-wise approach (stage 1 to stage 4), namely assessment, control, eradication, and post eradication stages. The PPR Monitoring and Assessment Tool (PMAT) is a companion tool to the PPR GCES to determine a country’s stage to provide guidance to countries. It takes into consideration the five technical elements identified in the GCES (Diagnostics, Surveillance, PPR Prevention and Control, Legal framework, and Stakeholder involvement). An analysis of PMAT reports from 32 countries found that most of the challenges faced by countries participating in the PMAT survey came under the element of “prevention and control” (46%). Based on experience from the successful global eradication of rinderpest and the availability of effective PPR vaccines, vaccination has been identified as the main prevention and control measure required for Stage 2 and Stage 3.

This paper reports on progress in vaccine development, PPR outbreaks under the vaccination campaign, vaccination campaign strategies, and vaccine management to gain experience to help guide the next decade of the GCES.

## 2. PPR Vaccine Development

After PPRV first emerged, rinderpest vaccines were used to cross protect against PPRV. Nigeria 75/1, a live attenuated vaccine for PPRV, was only introduced in 1989, and since that time, rinderpest vaccines have been banned for the prevention of PPR [32]. At present, the PPRV Nigeria 75/1 lineage II live attenuated vaccine has achieved mass production and has been used in all African countries, Middle Eastern, and Asian countries [33,34,35]. Due to the circulation of lineage IV in India and Asian countries, PPRV lineage IV was isolated (Sungri 96) in India in 1996 and passaged continuously for 59 generations on a marmoset lymphoblastoid cell line to obtain the live attenuated vaccine Sungri 96, which was shown to provide effective immunity for at least 6 years [34,36]. Experimental challenge studies have shown that the above two attenuated vaccines can induce protective immunity against all four genetic lineages of PPRV when challenged subcutaneously [37,38] or intranasally [38], which mimics the natural route of infection. The Sungri 96 vaccine has been used mainly in India and Bhutan.

PPRV inactivated vaccines currently under development, for example, Morocco/2008 PPRV inactivated vaccine developed by Cosseddu [39] and Morocco/2008 PPRV inactivated vaccine formulated with delta inulin adjuvant [40] are unlikely to provide the same lasting immunity as live attenuated vaccines, which limits their usefulness in endemic areas. However, although the current live attenuated PPRV vaccine has been used safely for decades, it has not been approved by Veterinary Authorities in non-endemic areas, such as Europe, because of the risk of reversion to virulence. In the non-endemic areas, inactivated vaccines are still the only alternative, and further research is needed [40].

The use of effective PPR live attenuated vaccines can induce lifelong protective immunity in vaccinated sheep and goats and cross-protect against all four PPRV lineages. The existing attenuated vaccines are widely used, but there are still some deficiencies, including low thermal tolerance and inability to differentiate infected from vaccinated animals (DIVA). Thermotolerant (TT) vaccines and DIVA vaccines need to be developed to facilitate the widespread use of PPR vaccines to enhance herd immunity and differentiate between vaccinated and naturally infected animals.

### 2.1. Progress in PPR TT Vaccines

Most PPR endemic countries are located in tropical or subtropical regions and have very limited cold-chain resources for vaccine storage and transport. None of the existing live attenuated vaccines are thermotolerant.

In 1999, Worrall et al. developed an ultra-rapid method using a formulation containing trehalose, called *Xerovac*, that dehydrates and preserves PPRV vaccines. This method can maintain a virus titer of the minimum acceptable titer (10^2.5^ TCID_50_/mL) under 45 °C for a period of 10 days [41]. In 2003, Sarkar et al. compared the stability of lyophilized vaccines using four stabilizers, namely hydrolyzed lactoprotein sucrose (LS), Weybridge medium (WBM), buffered gelatin–sorbitol (BUGS), and alginate dihydrate (TD). They recommended that LS be used as a lyophilized stabilizer and 1 M MgSO_4_ as a vaccine diluent to maintain the virus titer (10^2.5^ TCID_50_/dose) for up to 8 h at 37 °C and 7 h at 45 °C [34]. In 2009, Sen et al. used a cell culture medium containing heavy water-MgCl_2_ to passage the prototype Sungri/96 PPRV on Vero cells. Experiments showed that the deuterated vaccine can be exposed to 37 °C and 40 °C and maintain virus titers over 10^2.5^ TCID_50_/mL for 28 days [36]. In 2011, Silva et al. tried to use Tris/trehalose solution to improve the stability of the PPRV Nigeria 75/1 vaccines so that the vaccine could maintain a titer of 10^3.3^ TCID_50_/mL and 10^4^ TCID_50_/mL for 114 h at 37 °C and 12 h at 45 °C, respectively. In 2014, the national veterinary research institute of Ethiopia evaluated Silva’s formulation and found it was more effective at maintaining titers of the lyophilized vaccine than the WBM formulation. The national veterinary research institute of Ethiopia transferred Silva’s technology and used it to produce TT lyophilized vaccines [33,42,43]. In 2017, Mariner et al. applied the rinderpest freezing-drying method to the Nigeria 75/1 PPRV strain and used LS as a stabilizer. The shelf-life of the vaccine was ≥105 and 13.7 days at 37 °C and 56 °C, respectively [44]. To determine the minimum standards for TT vaccines, a Peste des Petits Ruminants Global Eradication Programme (PPR-GEP)-TT PPR Vaccines Workshop was organized in Rome (11–12 December 2017). The participants agreed that minimum requirements should be set for manufacturers to be able to use the term “TT vaccine”. The workshop provided temperature values and periods to be used as minimum requirements for thermotolerance (Table 1) after the dilution of freeze-dried vaccines with the diluents. It was, however, recognized that the minimum requirement levels for temperature and duration would need to be further validated based on market needs for the PPR GEP rather than on the technological capabilities and perspectives presented by researchers and vaccine producers at the meeting. The findings are awaited.

Thermotolerance of the freeze-dried vaccine before dilution is also very important in hot areas where the cold chain is not very reliable, and to allow people to transport it by road, as was done with the TT rinderpest vaccine. For an RP vaccine, it was considered TT when the vaccine was seen to be stable at 30 °C for 30 days. The same strategy is also planned for PPR vaccines, and experiments are ongoing to achieve the target as achieved for the RP vaccine.

### 2.2. Progress in PPR DIVA Vaccines

Traditional attenuated and inactivated vaccines do not enable the differentiation of infected from vaccinated animals. Recombinant vaccines that contain genetic markers can achieve DIVA goals.

In 2012, the first recombinant vaccine using a reverse genetics system to rescue PPRV was established, with a green fluorescent protein (GFP) marker [45]. In 2015, Muniraju et al. developed a vaccine engineered to lack the binding site of the C77 monoclonal antibody on the PPRV hemagglutinin (H) envelope glycoprotein [46]. From 2014 to 2017, Liu et al. generated a variety of PPRV virus-like particles (VLPs) by expressing different PPRV proteins [47,48,49]. Other recombinant vaccines using Capripoxvirus [50,51,52,53], Adenovirus [54,55,56,57,58], Vaccinia virus [59,60], Newcastle disease virus [61], or PPR [62,63] as vectors have been reported. To date, all of these recombinant vaccines do not provide the ability to differentiate wild type virus infection from vaccinal immunity and currently remain at an experimental stage. Some of these above vaccines were also evaluated successfully as multivalent vaccines in sheep and goats. Recently, two recombinant live attenuated PPR DIVA vaccines and DIVA ELISAs were developed at the Pirbright Institute, UK, and successfully evaluated in goats. Both the DIVA vaccines were safe and potent and provided similar immune responses as provided by their parent Nigeria 75/1 and Sungri 96 live attenuated vaccines [64].

A novel DIVA approach for PPRV was recently described, which does not require a DIVA-compatible vaccine nor the need for negative marker-specific serological monitoring technology [65]. Xue et al. designed a diagnostic hybrid protein–peptide microarray, which can discriminate the distinct IgG sero-dynamics against 4 epitope-containing short peptides (ECSPs) induced by vaccination or infection. The anti-ECSP IgGs of uninfected goats only existed 10–60 days post-vaccination. After 60 days post-vaccination, they detected positive anti-ECSP IgGs in 13 of 26 goats by the microarray, indicating that the flock was infected with the wild strain. This method can be used to differentiate infection by field PPRV strains from vaccination with live attenuated PPR vaccines. However, this microarray method may not be simple to adopt in National PPR laboratories and needs further validation in a large number of animals in the field.

## 3. PPR Outbreaks under the Vaccination Campaign

### 3.1. Global PPR Vaccine Use during 2015–2018

The objectives of vaccination campaigns for diseases, such as PPR (GCES), are that all small ruminants over 3 months of age should be vaccinated to reach a post-vaccination level of 70% immunity at flock, geographical area, or farming system level to break the epidemiological virus maintenance and spread cycle. According to the GCES, PPR vaccination should be implemented during two successive years, followed by vaccination of newborn animals during one or two successive years (FAO/OIE, 2015).

Understanding the status and effects of PPR vaccine use over recent years is helpful to evaluate vaccination programs and to adjust future prevention and control strategies. Between 2017 and 2021 of the first phase of the PPR Global Eradication Programme (PPR GEP), a target total of 1.5 billion small ruminants is planned to be vaccinated [66]. According to World Animal Health Information System (WAHIS), from 2015 to 2018, more than 333,016,000 doses of vaccine were used worldwide, with annual doses of 647,262; 28,258,654; 44,460,680; 91,242,883, respectively. Of these, 41.8% were used in African countries and 58.2% in Asian countries. Without considering the period, 2019–2021, and unreported vaccination campaigns, the above represents only 22% of the target vaccination set for 2021.

**Figure 1:**
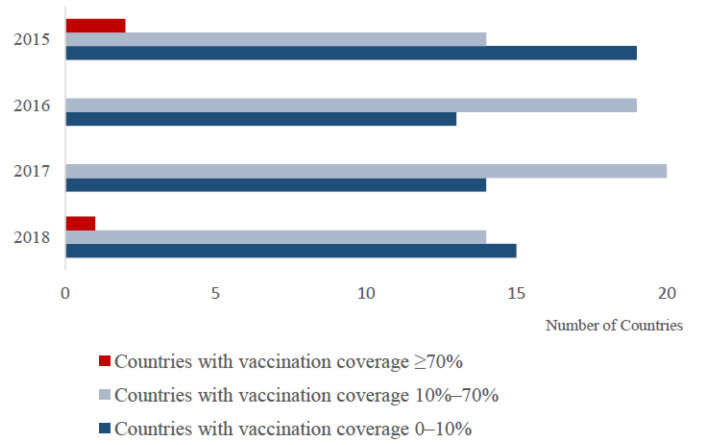
The number of countries with different vaccination coverage percentages for which data were available. Vaccination coverage is estimated by dividing the number of vaccine doses used (derived from WAHIS) by the number of goats and sheep in the country (derived from FAOSTAT).

The following is an overview of vaccination from 2015 to 2018. The number of countries with different vaccine coverage rates is shown in Figure 1. Due to the lack of data on post-vaccination sero-monitoring, the data shown are for vaccination coverage (i.e., % small ruminant population vaccinated). However, clearly, the proportion of animals with protective immunity following vaccination is the key parameter for disease control. Therefore timely post-vaccination sero-monitoring is needed to assess the efficacy of vaccination campaigns.

### 3.2. PPR Outbreaks from 2015 to 2019

**Figure 2:**
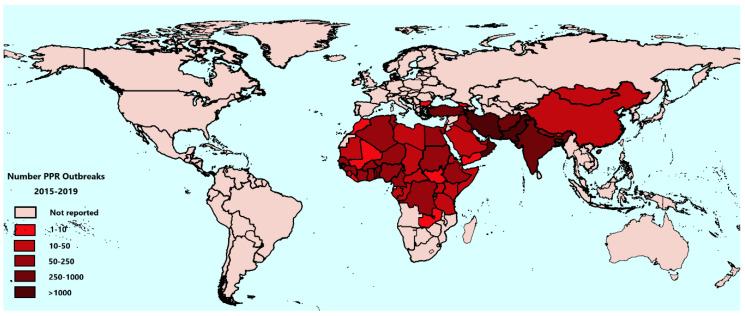
Global distribution of Peste des petits ruminants (PPR) burden showing the total number of reported PPR outbreaks from 2015 to 2019. Data are taken from the World Animal Health Information System (WAHIS).

The analysis of PPR outbreaks in recent years helps reveal the latest epidemiological situation of PPR globally and clarify the effects of PPR control in various countries. The total number of PPR outbreaks from 2015 to 2019 in countries is shown in Figure 2, using WAHIS data. Between 2015 and 2019, a total of 12,757 outbreaks were reported to the OIE by 59 countries, out of 66 recorded to be infected, indicating that PPR may be under-reported in the remaining 7 infected countries. Among them, 9582 outbreaks were in Asia and the Middle East, accounting for 75.1%, and 3166 outbreaks were in Africa, accounting for 24.8%, 9 outbreaks were in Europe (Bulgaria only), accounting for 0.1%. The distribution of outbreaks between the nine global sub-regions defined within the PPR GCES is shown in Table 2. In 2015, the number of PPR outbreaks worldwide was 3688; from 2016 to 2018, there were 2858, 2405, and 2588 outbreaks, respectively (WAHIS data). In 2019, the number of reported PPR outbreaks showed a marked decrease to 1218. Since 2015, the number of PPR outbreaks worldwide has been decreasing, and the number of PPR outbreaks in Cameroon, Afghanistan, Kuwait, and Oman has decreased year by year.

When verifying their PPR free status, countries need to confirm that there have been no PPR outbreaks and no evidence of PPRV infection in the past 24 months and that vaccines were not used during the same period. Currently, 21 of the 66 countries classified as infected have had no reported PPR outbreaks for more than 24 months, and 10 of these have had no outbreaks between 2015 and 2019, namely Angola, Bahrain, Jordan, Kazakhstan, Kyrgyzstan, Lebanon, Qatar, Syrian Arab Republic, Tajikistan, and Uzbekistan.

Notably, the number of outbreaks in many countries is high: 22 countries suffered more than 100 outbreaks between 2015 and 2019, including 15 in Africa and 7 in Asia. Of these, more than 400 PPR outbreaks have been reported in 5 countries, namely Benin (480), Afghanistan (824), Iran (3710), Kuwait (761), and Turkey (402). From 2015 to 2019, PPR outbreaks in these five countries accounted for 48.4% of all global outbreaks. In addition, there has been an increase in reported outbreaks in 2018 and 2019 in Algeria, Ethiopia, and Guinea. Therefore, it is necessary to concentrate on solving the difficulties encountered by these countries in prevention and control and adjust national strategies according to their current situation.

## 4. Vaccination Strategies

### 4.1. PPR Vaccination Strategies in Countries from 2015 to 2018

Over recent years, vaccination has been carried out in almost all PPRV-infected countries. Countries have adopted a range of approaches for vaccination depending on their epidemiological situation and the extent to which they have targetted their vaccination strategically according to the disease situation and their National Strategic Plans. Examples of the vaccination coverage reported by countries during Regional Roadmap Meetings are shown in Table 3.

In the subsequent sections, data marked with asterisks (*) are from countries’ reports, and conclusions may be constrained by the reliability of the data.

#### 4.1.1. Targeted Cost-Effective Vaccination Strategies Can Be Used to Maintain Uninfected Status or Control Sporadic Cases

Between 2015 and 2018, Lebanon conducted a vaccination campaign only in 2015. In contrast, Jordan, Kazakhstan, and Kyrgyzstan have been vaccinating annually from 2015 to 2018, sometimes in border areas, and with <70% vaccine coverage nationally. They are considered to be in stage 2 or 3.

As a result of control measures implemented after the first outbreak in mid-July 2008, PPR was absent from Morocco from 2009, but it re-emerged in the country at the end of June 2015. To clear its sporadic cases, Morocco launched a mass vaccination campaign with 86%* vaccine coverage in 2015, which lead to no new outbreaks occurring since then. In Burundi, PPR was first detected in December 2017, and 8 outbreaks occurred in the country in 2018. Burundi implemented two national vaccination campaigns in 2018 and 2019, and seropositivity rates of 91% (2018) and 98% (2019) were achieved (personal communication; Felix Njeumi). The estimated population of sheep and goats in Georgia is approximately 900,000. After three cases were reported in 2016, Georgia implemented mass vaccination in 2016, 2017, and 2018 with 1,650,974*, 341,461*, and 339,655* vaccine doses used, respectively. As a result, no new cases have since been reported.

Unlike Morocco, Burundi, and Georgia, after a small number of outbreaks were identified within a limited area, South Sudan has only conducted vaccination with low vaccine coverage in 2016 (vaccine coverage 0.6%) and 2017 (vaccine coverage 0.9%). No new outbreaks have been reported since 2018.

In countries where outbreaks are rare, targeted vaccination may enable the elimination of the disease in a cost-effective manner.

#### 4.1.2. Vaccination Coverage May Not Be Sufficient to Achieve PPR Elimination

In some countries, despite high vaccination coverage, immunity was not sufficiently high to stop the virus circulation. For example, Saudi Arabia and Iraq did not show a significant decrease in the number of outbreaks despite their high vaccination coverage. In Saudi Arabia, PPR was suspected clinically in sheep in the 1980s. However, no virus was isolated until 1990, when the virus was successfully isolated during an outbreak in indigenous goats. Since then, PPR is endemic in many regions in the kingdom of Saudi Arabia with continuing outbreaks, especially during the cold season. Sheep and goats are two main sources of meat production in the Kingdom of Saudi Arabia, with estimated populations of about 14,500,000 and 4,500,000 head, respectively. In 2015, about 357* PPR outbreaks were reported, involving 33,169* suspected infected animals. In 2015, around 8.8 million head were vaccinated, and between 2015 and 2019, 45,567,948 doses of vaccine have been used. Compared to the country’s small ruminant population, the vaccination rate was still very low. As a result, outbreaks have continued in recent years. Iraq is in stage 2 of the stepwise approach of the GCES. Although vaccination coverage is high in Iraq (Table 3), unfortunately, sero-monitoring has never been carried out. During the same period, clinical cases reported by veterinary services were 111 in 2014*, 410 in 2015*, 125 in 2016*, 412 in 2017*. From 2015 to 2019, PPR outbreaks have occurred every year, and in total, 83 outbreaks in Iraq have been recorded by OIE.

PPR was diagnosed for the first time in sheep, goats, and yaks during September 2016 in the western region of Mongolia. Measures, such as rapid vaccination and restriction of livestock movement, have significantly reduced the spread of the disease. Between 2016 and 2019, focused vaccination was carried out in western and eastern regions of the country with 10.2 million, 4.8 million, 14.2 million, and 5.3 million small domestic ruminants vaccinated, respectively, totaling 34.5 million head. The last reported outbreak was in 2017. In western Mongolia, between 2017 and 2019, vaccination was targeted only for offspring. Following the first reports in livestock, PPR was also diagnosed in wild ungulates, including the critically-endangered Mongolian saiga antelope, Siberian ibex, goitered gazelle, and argali (wild sheep) in January 2017 [19,20]. Epidemiological evidence suggests spill-over of PPRV from livestock and subsequent spread among wild ungulates [17].

#### 4.1.3. Some Countries with Many PPR Outbreaks due to Lack of Sufficient or Timely Vaccine Coverage

More than 20% of infected countries, including Benin, Burkina Faso, the Republic of the Congo, Ghana, Guinea, Guinea-Bissau, India, Kuwait, and Nigeria, are suffering from PPR outbreaks but deploy vaccine doses equivalent to <10% of the total goat and sheep populations on an annual basis. For example, in Burkina Faso, from 2015 to 2018, outbreaks occurred continually. The total number was over 130, and the small-scale vaccination equates to only 2.5%, 1.9%, 1.8%, and 0.9% of the national sheep and goat population, respectively.

The Democratic Republic of the Congo, Tanzania, Sierra Leone may not have taken timely action to vaccinate following outbreaks since no vaccination was reported. The sheep and goat population in the Democratic Republic of the Congo was 5,002,000 (2016), and the total number of outbreaks from 2015 to 2019 was more than 160. However, from 2016 to 2019, OIE has no record of vaccine use in the Democratic Republic of the Congo. However, data presented by the country’s representative at the road map meeting showed that in October 2016, a post-vaccination evaluation was carried out in a few regions: Bas- Congo (67.4%), Bandundu (50.3%), Maniema (32.3%), and Kasai Occidental (16.4%). Cases have continuously been reported in Tanzania from 2015 to 2019, but no reported vaccination was conducted between 2015 and 2017. In 2019, 452,339 animals were vaccinated, representing 1.7% of the national flock. In Sierra Leone, the small ruminant population was approximately 2,183,836*, and 41,202*doses of vaccine were used in 2015. PPR is still endemic in 6 of the 16 districts in the country, with a mortality rate of 9.3–16.5%*, and no vaccination was undertaken after outbreaks in 2018. Similarly, high disease burdens, but the absence of vaccination is seen for Guinea-Bissau and Kuwait.

### 4.2. Research Advances to Inform Evidence-Based PPR Control and Vaccination Strategies

A study in Tanzania analyzed PPRV transmission risk by age and type of production system based on serum survey data collected in 2016. It was found that the PPRV seropositivity rate of sheep, goats, and cattle did not change significantly with age. However, the serum antibody positive rate of pastoral animals at all ages was significantly higher than that of agropastoral animals. This study suggests that targeting control based on animal age may not be as effective as targeting other risk factors, such as the type of production system [67].

To understand the reasons for low PPR vaccination rates among livestock in Mali, Wane et al. (2019) surveyed 304 farmers and found that 89% of farmers had their livestock vaccinated during the official vaccination campaign. Farmers’ awareness of the benefits of vaccines and faster access to vaccination information is beneficial to the implementation of official vaccination campaigns [68]. Only 39% of respondents had private vaccinations outside official vaccination campaigns. In addition to the farmers’ awareness, their trust in veterinarians is beneficial for private vaccination, and fears of side effects reduced farmers’ willingness to use the vaccine. The results of this study can better guide the management of vaccination and thus increase the willingness of farmers to use PPRV vaccines [68].

Senegal used the evaluation of livestock vaccination costs tools, ‘VacciCost’, and estimated the resources needed to implement a mass vaccination campaign of livestock [69]. This decision-making tool considers eight categories of resources (i.e., vaccines, injection supplies, transport, personnel, training, maintenance and overheads, social mobilization, surveillance, and monitoring) as well as different livestock production systems that influence the operational difficulty of vaccinating. Vaccination productivity, i.e., the number of animals vaccinated per day by vaccination teams, was found to be crucial in determining the net costs of the campaign. When the same amount of vaccine is used, low productivity will result in a significant increase in personnel costs, and since training accounts for only 1% of the total cost (under all scenarios investigated), significant savings may be achieved through training aimed at increasing the productivity of vaccination teams [69]. Countries may use the VacciCost tool to estimate the resources required for PPR vaccination, design cost-reduction strategies, and help prepare national budgets for PPR control and eradication.

To maximize the economic benefits of PPR control and eradication, vaccination programs require careful planning using scientific evidence. In India, three axes of PPR mass vaccination campaign, namely, adequacy (vaccination coverage, number of outbreaks, diagnosed and death cases, and vaccination seroconversion), financial viability (program under different scenarios and options), and the program impact from farmers’ perspective were evaluated [70]. The study showed that the reported outbreaks, diagnosed and death cases declined in consonance with increased vaccination coverage. The study found that a strategy of vaccinating 100% of the at-risk population in the first year and 30% of the naïve population in the subsequent years would yield more benefits than vaccinating 100% coverage every year. Regardless of whether 30% or 100% of animals were vaccinated in subsequent years, the benefits outweighed the costs. The results provide a reference for the implementation of a mass vaccination campaign in India or other countries with similar socioeconomic environments and small ruminant production systems [70].

## 5. Management of PPR Vaccination

### 5.1. Implementation of Vaccination Campaigns

Some recent vaccination campaigns provide important lessons to guide the implementation of vaccination in other countries and regions. For example, in 2012, approximately 20 million sheep and goats, constituting 60% of the estimated national small ruminant population of Somalia, were vaccinated throughout Somalia. This was led by the FAO using a private–public partnership approach. Before the vaccination campaign, cold chain facilities were placed in strategic locations where vaccines were stored. Two serological surveys were conducted before and after the vaccination campaigns, where a two-stage cluster sampling methodology was used to collect 20,000 sera samples for analysis. The results showed an overall increase in individual animal seroprevalence from 62 to 76% after the PPR vaccination campaign [71]. As a result, about 25 outbreaks were reported to OIE from 2015 to 2019.

A pilot vaccination strategy against PPR that was implemented in Burkina Faso and Ghana between 2013 and 2014 proved successful in producing protective immunity in the PPR virus in a profitable manner. Four different vaccination protocols were tested: (1) no vaccination, (2) free PPR vaccine without contribution to operational costs, (3) free PPR vaccine with a partial contribution to the operational costs, (4) free PPR vaccine with a partial contribution to the operational costs and free distribution of anthelmintics. The third protocol proved the most effective. Difficulties that were determined through this study included the importance of appropriately timing vaccinations, educating farmers on small ruminant management, and providing identification to vaccination teams for farmers to identify them. In addition, key components that were identified as critical for successful vaccination included community awareness, appropriate duration of the vaccination campaign, and proper handling of the vaccines during this time, and a good relationship/communication between livestock owners and veterinary services [72].

In 2015–2020, during the implementation of the Regional Sahel Pastoralism Support Project (PRAPS) project, 72.8 million doses of PPR vaccine were ordered for Africa through the OIE Vaccine Bank. Since 2019, approximately 100 million animals have been vaccinated by FAO in Afghanistan, Burundi, the Central African Republic, Côte d’Ivoire, Ethiopia, Guinea, Kenya, Mali, Niger, Pakistan, Sierra Leone, Somalia, South Sudan, Sudan, Syria, and Yemen. These vaccines were stored in United Nations storage facilities or shipped directly to countries following international procurement processes. In addition, these vaccines need to be quality assured by the African Union Pan African Veterinary Vaccine Centre (PANVAC) recognized to ensured quality control of PPR vaccines globally. Before the vaccination campaign, the recipient countries ensured a cold chain system was in place so that adequate temperature control was secured from the laboratory to the injection of the animal.

### 5.2. Challenges for the Distribution and Use of PPR Vaccines

Stakeholders at all levels have facilitated affordable access to quality-assured vaccines manufactured in line with OIE standards to ensure countries can procure high quality, timely, and affordable vaccines. However, based on an analysis of available PMAT questionnaires from 32 countries collected during road map meetings, among the various challenges under “Prevention and Control”, shortage of vaccines is still reported as one of the three most prominent factors, the others being illegal or legal livestock movement and lack of resources for logistics of the vaccination campaign.

Improper distribution of vaccines may be one of the reasons for the shortage of vaccines, and underlying the issue of vaccine distribution are data and infrastructure gaps. According to the PMAT report from 32 countries, the top three challenges faced by countries under the category of ‘Surveillance systems’ include lack of an established system and information (7 countries), lack of epidemiological understanding (7 countries), and inadequately estimated livestock number (3 countries). Poor capacity for data collection and analysis will impede the rational distribution and the epidemiologically-informed deployment of vaccines.

Comparing the data from the public data-sharing platforms FAOSTAT and OIE-WAHIS with national reports made during road map meetings, inconsistencies are evident. The data difference may be caused by different data sources and reporting time. Data on livestock numbers in FAOSTAT are calculated based on “Guidelines for the preparation of livestock sector reviews” (FAO, 2011), while livestock numbers in OIE-WAHIS are reported by country/territory. The data from OIE-WAHIS are updated periodically, for example, information on vaccines are reported by country/territory once per year, whereas roadmap meeting reports contain the most recent data. However, many countries are not reporting in a timely manner, and data in roadmap meeting reports are often missing relevant information, including key data on collection date and sample size, etc., which leads to poor data availability and reliability.

Consultations with a broad range of stakeholders through regional workshops and vaccine producers’ meetings have also identified the need for greater quality and quantity of currently produced vaccines. Shortages in vaccine production, inadequate quality assurance, or inability to deliver the vaccines to meet field needs represent risks to the planned campaigns and constitute a major challenge to progress in the time-bound PPR-GEP. Other challenges were identified during the PPR vaccine producers meetings (Kathmandu (2014), Morocco (2017), and Amman (2019) organized by FAO and OIE): (i) producers do not provide diluents leading to the use of a variety of non-standard products (which might potentially impact vaccine efficacy); (ii) lack of information about the perceptions of farmers about vaccination, (iii) countries’ demands for PPR vaccine are not known, so manufacturers cannot predict potential volume requirements, (iv) lack of cold chain facilities, (v) current vials are for 100 doses, whereas there is an unmet need for smaller dose vials is some areas.

### 5.3. Funding Gaps for Operationalising Effective Vaccination Campaigns Still Remain

The first phase of the PPR program set a target of vaccinating 1.5 billion small ruminants by the end of 2021. As per mid-2020, around half of this target had been achieved. The funding gap required for vaccine procurement and to implement vaccination campaigns, including equipment and manpower, is delaying the use of the vaccine in many countries. For example, Kenya faces a shortage of personnel and infrastructure, and the Democratic Republic of the Congo has also raised the issue of a lack of logistical equipment, which has affected PPR control. The cost of one vaccine dose represents around 1/8th of the cost of vaccine delivery. The lack of logistical resources for vaccination campaigns is one of the three most prominent challenges hindering “prevention and control” of PPR, according to the PMAT report.

## 6. Conclusions

The total number of PPR outbreaks worldwide has decreased significantly in recent years, but the infection scope of PPRV, both geographical and host range, is still wide. Remarkably, more than 48% of all outbreaks between 2015 and 2019 have occurred in 5 countries, highlighting the urgent need for these countries to strengthen prevention and control. Twenty-one countries, which have had no new cases for five consecutive years, can prepare their documentation for OIE validation of their PPR free status.

Countries adopted different strategies in implementing vaccination campaigns from 2015 to 2018. Vaccination had a positive effect in some countries, encouraging them to maintain their status of no new cases being reported. Implementation of vaccination campaigns in many countries with recurrent or new outbreaks can lead to the elimination of PPR in a short period. In most countries, however, the epidemiological situation is more severe and complex, and the epidemics of PPR have not been significantly alleviated following vaccination.

According to data obtained by FAOSTAT and WAHIS, there is a clear disparity in vaccine coverage, with some countries using vaccine doses close to their total ruminant population in a single year, while vaccine coverage was less than 10% in several severely infected countries. In both cases, the vaccination-induced immunity needs to be increased further. This indicates the need for an evidence-based assessment of vaccination strategies, including vaccination cycles and vaccine coverage, to improve the effectiveness of disease control and avoid waste of resources. The development of user-friendly and convenient tools to optimize data collection and reporting and strengthen the management of monitoring system data will have a positive impact on the timely adoption of prevention and control measures and the most cost-efficient allocation of resources.

In addition, the funding gap for vaccines and logistics of vaccination campaigns is one of the most important factors affecting prevention and control. It must be addressed through dialogue between organizations and countries, resource mobilization, and rationalization of funding.

## Figures and Tables

**Table 1:** Temperature values and time periods to be used as minimum requirements for thermotolerance, as recommended at the Peste des Petits Ruminants Global Eradication Programme (PPR-GEP) thermotolerant (TT) vaccines workshop. These values refer to storage after dilution of the freeze-dried vaccines with diluent.

Storage	Standard Cold Chain	Room	Field
Temperature	2–8 °C	25 °C	40 °C
Time Period	2 years	10 days	5 days

**Table 2:** Peste des petits ruminants (PPR) outbreaks reported to the World Organization for Animal Health (OIE) from 2015 to 2019 for nine different sub-regions.

Sub-Regions	No. of Outbreaks	Proportion of Total Reported Outbreaks Globally
Southern Africa	171	1.3%
Central Africa	199	1.6%
Western Africa	1912	15.0%
Eastern Africa	485	3.8%
Northern Africa	394	3.1%
Middle East	7710	60.4%
West Eurasia	405	3.2%
South Asia	1386	10.9%
East Asia	58	0.5%
Countries not included in the Nine sub-regions	37	0.3%

**Table 3:** Countries’ reports of vaccination coverage during road map meetings. Numbers show the percentage of the small ruminant population vaccinated.

Country	2010	2011	2012	2013	2014	2015	2016	2017	2018
Eritrea						7.7			
Iraq		73.5	77.6	91.0	89.8		78.9	78.5	67.8
Kazakhstan						57.0			
Tajikistan	16.0	8.6	3.2	5.2	21.9	10.0			
Turkey	44.7		82.1	77.9	79.0	69.3			
South Sudan		1.2	0.8	0.9	1.5				
Sudan			15.5	12.8	39.7				
Uganda		10.5							
